# Impact of coronary revascularization on clinical outcomes in vessels with discordant results of fractional flow reserve and resting full-cycle ratio

**DOI:** 10.1007/s00380-025-02605-8

**Published:** 2025-09-27

**Authors:** Tatsuro Yamazaki, Yuichi Saito, Shunsuke Nakamura, Yuya Tanabe, Hideki Kitahara, Yoshio Kobayashi

**Affiliations:** Department of Cardiovascular Medicine, Chiba University Graduate School of Medicine, 1-8-1 Inohana, Chuo-ku, Chiba, Chiba 260-8677 Japan

**Keywords:** Fractional flow reserve, Resting full-cycle ratio, Discordant result, Coronary revascularization

## Abstract

Fractional flow reserve (FFR) is an invasive standard, and resting full-cycle ratio (RFR), a non-hyperemic pressure ratio, is an alternative to FFR for evaluating the functional severity of coronary stenosis. However, the prognostic impact of coronary revascularization in vessels with discordant results of FFR and non-hyperemic pressure ratios remains unclear. This single-center study included 212 vessels in 191 patients with intermediate coronary stenosis and discordant results of FFR and RFR. FFR ≤ 0.80 and RFR ≤ 0.89 were considered physiologically positive. Vessels with discordant results of FFR and RFR were divided into two groups according to the revascularization strategies—the deferral and revascularization groups. The primary endpoint was target vessel failure (TVF), a composite of cardiac death and target vessel myocardial infarction and unplanned revascularization. Of the 212 vessels, 145 (68.4%) and 67 (31.6%) were categorized as the deferral and revascularization groups, respectively. The deferral group was more likely to be older and women than the revascularization group. FFR values were higher, and the rate of positive FFR was lower in the deferral group than in the revascularization group. During the median follow-up of 406 days, 12 of 212 (5.7%) developed TVF. The Kaplan–Meier analysis demonstrated that the TVF rate was significantly lower in the revascularization group than the counterpart (7.6% vs. 1.5% at 3 years, P = 0.046). In conclusion, coronary revascularization in vessels with discordant results of FFR and RFR was associated with lower TVF rates as compared with the deferral strategy.

## Introduction

Fractional flow reserve (FFR), a ratio of mean distal coronary pressure (Pd) and mean aortic pressure (Pa) during maximal hyperemia, is widely recognized as an invasive standard to assess the functional severity of epicardial coronary artery stenosis [1–3]. In the international guidelines for chronic coronary artery disease (CAD), FFR has been recommended for guiding the clinical decision-making for coronary revascularization [1–3]. Based on the principle, FFR is evaluated in a hyperemic condition, while non-hyperemic pressure ratios (NHPRs), which have emerged as an alternative to FFR for assessing the functional severity of epicardial CAD, are measured in a resting condition [1–3]. Two large-scale randomized control trials showed that instantaneous wave-free ratio (iFR), one of the NHPRs, was non-inferior to FFR in guiding coronary revascularization [4, 5]. Subsequently, the guidelines recommend NHPRs as well as FFR for assessing the functional severity of intermediate coronary artery stenosis [1–3]. To date, other NHPRs than iFR have been developed, such as resting full-cycle ratio (RFR) and diastolic pressure ratio, which can be used in clinical practice with the same cut-off value of 0.89 for estimating FFR ≤ 0.80 [6, 7]. However, it has been reported that FFR and NHPR have discordant results in approximately 20% of lesions with angiographically intermediate CAD when using their standard cut-off values recommended in the guidelines (i.e., FFR ≤ 0.80 and NHPRs ≤ 0.89) [1–3, 8–10]. Although several studies have shown that vessels with discordant results between FFR and NHPRs had a higher risk of adverse cardiovascular events than those with concordantly negative results, whether revascularization can reduce coronary events in such lesions and vessels is still controversial [7, 10–12]. Thus, we aimed to evaluate the potential benefit of coronary revascularization on clinical outcomes in vessels with discordant results of FFR and RFR.

## Methods

### Study population

**Fig. 1:**
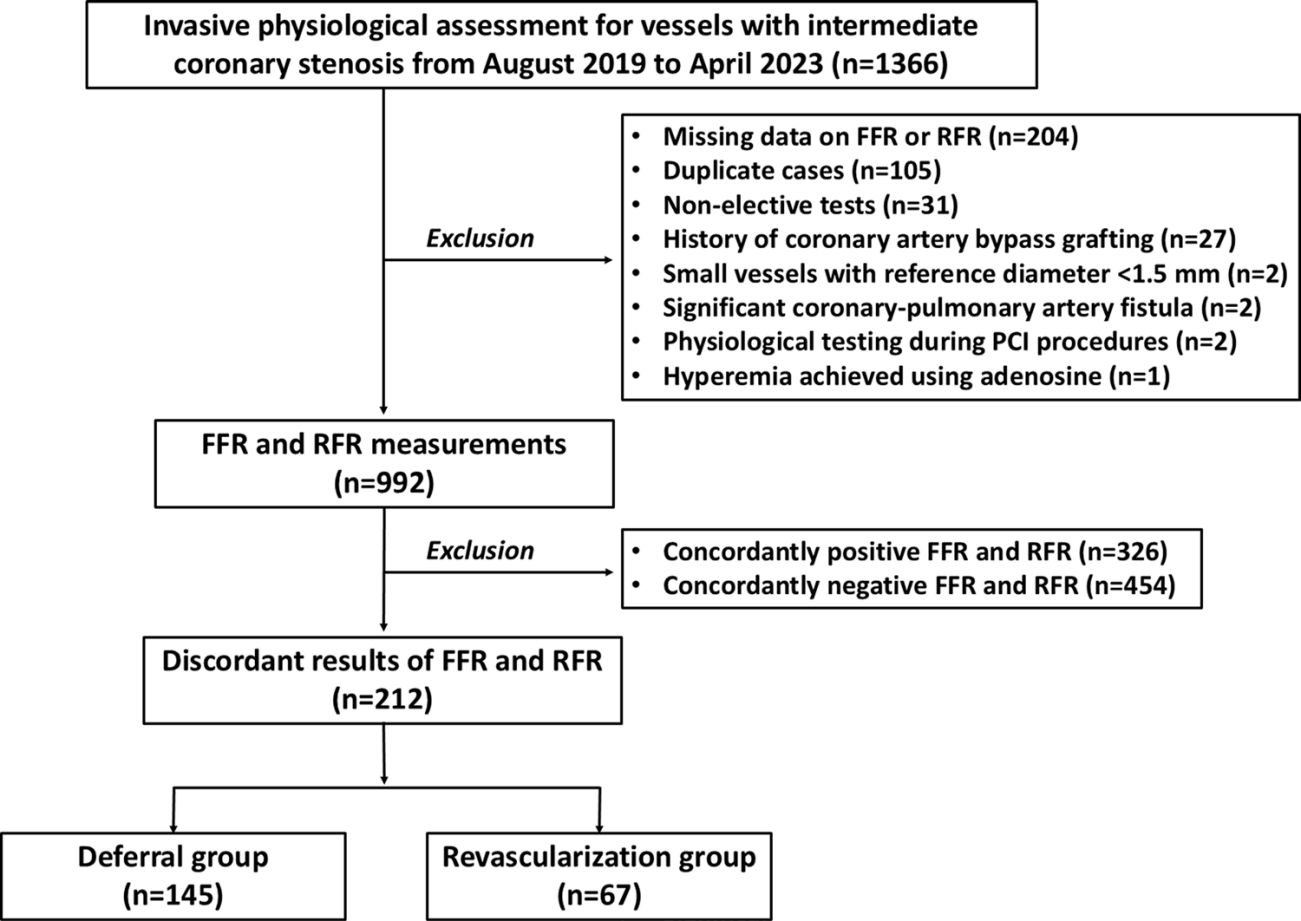
Study flow. *FFR* fractional flow reserve; *PCI* percutaneous coronary intervention; *RFR* resting full-cycle ratio

This was a single-center, retrospective, observational study done at Chiba University Hospital. A total of 1366 vessels in 890 patients with intermediate epicardial coronary stenosis underwent invasive physiological testing from August 2019 to April 2023 (Fig. 1). Vessels with any missing data on FFR or RFR, duplicate cases, physiological assessment in a non-elective setting, a history of coronary artery bypass grafting, small vessels with reference diameter < 1.5 mm, significant coronary-pulmonary artery fistulas, physiological testing during a percutaneous coronary intervention (PCI) procedure, and maximum hyperemia achieved using intravenous administration of adenosine were excluded (Fig. 1). This study adhered to the Declaration of Helsinki and was approved by the institutional ethics committee of Chiba University Hospital. Informed consent for the present study was ascertained in an opt-out manner.

### Coronary physiological assessment

Physicians performed coronary angiography from the radial, brachial, or femoral artery with a 4 to 6 Fr catheter per local standard protocol [13, 14]. Coronary physiological assessment was conducted in vessels with intermediate coronary artery stenosis during angiography at the operator’s discretion. Equalization of Pa and Pd was confirmed at the ostium of the coronary artery and maximum hyperemia was achieved by intracoronary administration of papaverine (8 mg for the right coronary artery and 12 mg for the left coronary artery) or nicorandil (2 mg) [13, 15, 16]. The presence of pressure drift was checked at the ostium of the coronary artery after physiological testing. When a significant pressure drift (≥ 0.03) was observed, physiological indices were re-evaluated. In this study, FFR ≤ 0.80 and RFR ≤ 0.89 were considered positive [1–3, 10]. Vessels with positive FFR and RFR were classified as being concordantly positive (i.e., FFR ≤ 0.80 and RFR ≤ 0.89) and those with negative FFR and RFR were classified as being concordantly negative (i.e., FFR > 0.80 and RFR > 0.89). Vessels with dichotomous disagreement between FFR and RFR were classified as the discordant group (i.e., vessels with FFR ≤ 0.80 and RFR > 0.89 or those with FFR > 0.80 and RFR ≤ 0.89). After excluding vessels with concordantly positive results (n = 326) and concordantly negative results (n = 454), vessels with discordant results of FFR and RFR were included in the present analysis and divided into two groups according to whether coronary revascularization was performed (Fig. 1). Therapeutic strategies were left to each operator’s discretion based on the guidelines in a real-world clinical practice setting. In this study, vessels in which coronary revascularization was not performed were defined as the deferral group, and their counterparts were defined as the revascularization group (Fig. 1).

### Primary endpoints

The primary endpoint of this study was target vessel failure (TVF), defined as a composite of cardiovascular death, target vessel myocardial infarction (MI), and unplanned target vessel revascularization [17, 18]. The primary interest of the present study was to compare the cumulative TVF risk between the deferral and the revascularization groups. As a sensitivity analysis, the cumulative TVF risk between the two groups was evaluated after excluding patients with severe aortic stenosis and maintenance hemodialysis. Follow-up data were obtained from medical records at Chiba University Hospital. In the present study, cardiovascular risk factors such as hypertension, diabetes, dyslipidemia, and current smoking were defined according to the Japanese Association of Cardiovascular Intervention and Therapeutics criteria [19, 20]. Chronic kidney disease is defined as an estimated glomerular filtration rate of less than 60 ml/min/1.73 m^2^. Because aortic stenosis and hemodialysis were strong factors associated with the discordant physiological results [8, 21, 22], the cumulative TVF risk was evaluated, as a sensitivity analysis, after excluding such patients.

### Statistical analysis

Contentious variables were expressed as mean ± standard deviation or median [interquartile range]. Student’s *t* test or Mann–Whitney U test was performed to compare the contentious variables between two groups, as appropriate. Categorical variables were represented as n (%) and were assessed with Fisher’s exact test. Kaplan–Meier analysis with the log-rank test was conducted to compare the cumulative risk of TVF between the deferral and revascularization groups. All statistical analyses were performed with EZR version 1.55 (Saitama Medical Center, Jichi Medical University, Saitama, Japan), which is a graphical user interface for R version 2.7.1 (The R Foundation for Statistical Computing, Vienna, Austria). A value of P < 0.05 was defined as being statistically significant in this study.

## Results

**Fig. 2:**
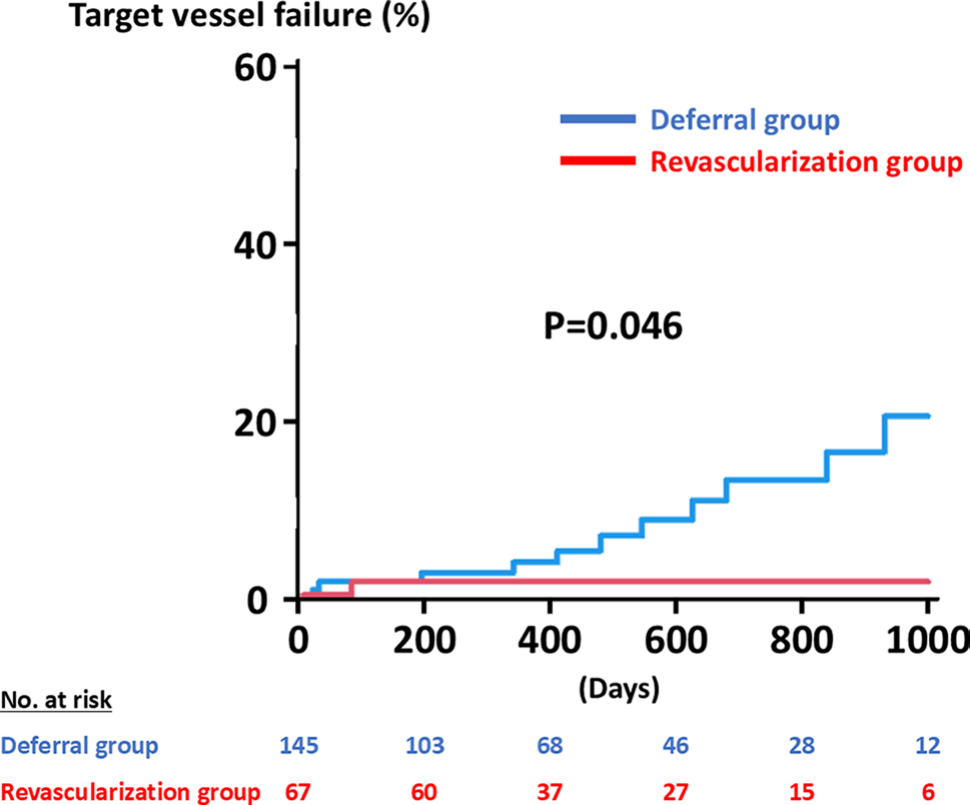
Comparative incidence of target vessel failure between the deferral and the revascularized groups

**Fig. 3:**
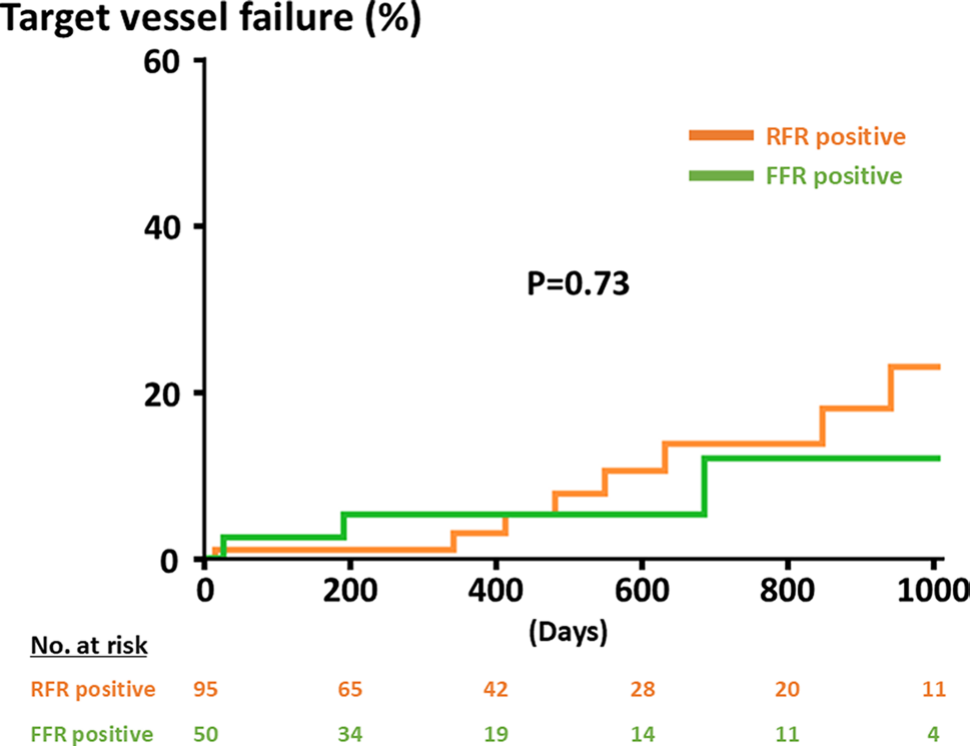
Comparative incidence of target vessel failure between positive FFR and RFR in vessels with discordant physiological results. *FFR* fractional flow reserve; *RFR* resting full-cycle ratio

**Fig. 4:**
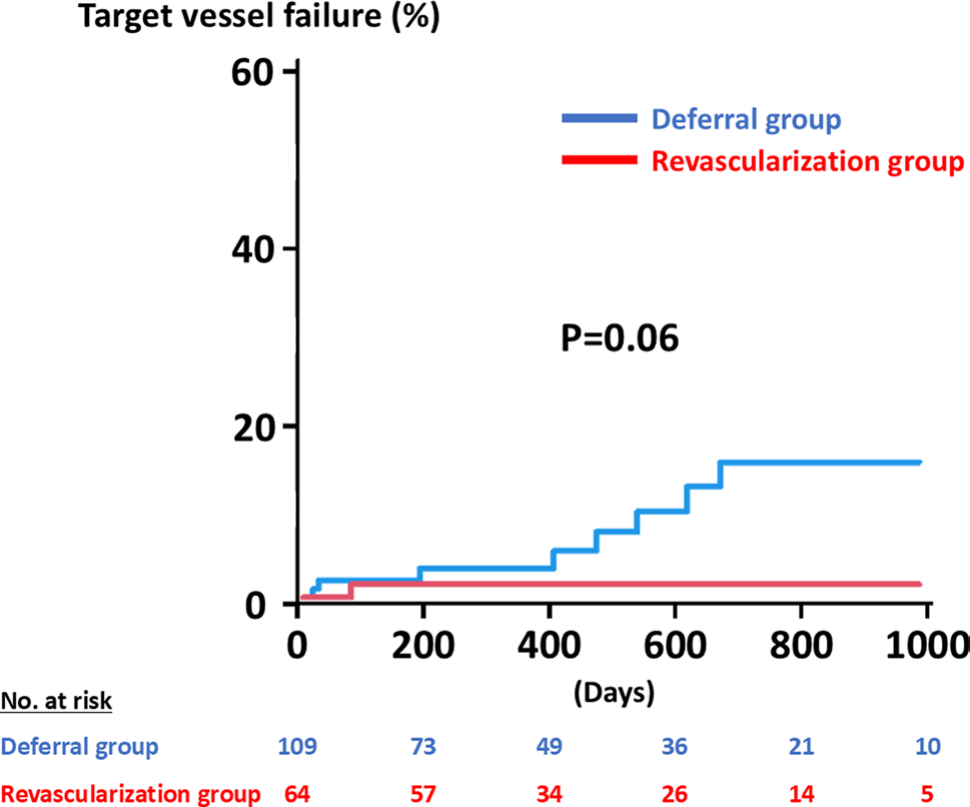
Comparative incidence of target vessel failure between the deferral and the revascularized groups after excluding patients with severe aortic stenosis and hemodialysis

A total of 212 vessels in 191 patients were included in the present analysis. Of the 212 vessels, 145 (68.4%) and 67 (31.6%) were categorized as the deferral group and the revascularization group, respectively (Fig. 1). Baseline characteristics are summarized in Table 1. The deferral group was associated with older age and a higher prevalence of women, chronic kidney disease, and hemodialysis, severe aortic stenosis, and anemia. Body mass index and rates of current smoking also differed significantly between the two groups (Table 1). Table 2 shows the physiological findings. Overall, mean FFR and RFR values were 0.81 ± 0.05 and 0.89 ± 0.05, respectively. Positive FFR and RFR were observed in 105 (49.5%) and 107 (50.5%) vessels. The deferral group had a significantly higher FFR value and a lower rate of positive FFR as compared to the revascularization group (Table 2). On the other hand, the RFR value was lower and the rate of positive RFR was higher in the deferral group than the counterpart (Table 2). During a median follow-up period of 406 days, 12 (5.7%) TVF events occurred (Table 3). The Kaplan–Meier analysis demonstrated that the cumulative risk of TVF was significantly higher in the deferral group than in the revascularization group (P = 0.046) (Fig. 2). No significant differences were observed in TVF risks between vessels with positive FFR and RFR (Fig. 3). After excluding vessels with severe aortic stenosis and hemodialysis, 173 were included in the sensitivity analysis. Baseline characteristics, physiological findings, and clinical outcomes are summarized in Tables 4, 5 and 6. The Kaplan–Meier analysis illustrates a similar trend to those in the entire study population (Fig. 4).

**Table 1:** Baseline characteristics

	All (*n* = 212)	Deferral group (*n* = 145)	Revascularization group (*n* = 67)	*P* value
Age (years)	72.9 ± 8.7	74.0 ± 8.8	70.4 ± 7.9	0.005
Men	161 (75.9%)	103 (71.0%)	58 (86.6%)	0.02
Body mass index (kg/m^2^)	23.6 ± 3.9	23.2 ± 3.8	24.4 ± 4.0	0.03
Body surface area (m^2^)	1.67 ± 0.20	1.63 ± 0.19	1.75 ± 0.19	< 0.001
Hypertension	167 (78.8%)	111 (76.6%)	56 (83.6%)	0.28
Diabetes	89 (42.0%)	62 (42.8%)	27 (40.3%)	0.77
Dyslipidemia	144 (67.9%)	97 (66.9%)	47 (70.2%)	0.75
Current smoking	35 (16.5%)	18 (12.4%)	17 (25.4%)	0.03
Hemodialysis	21 (10.0%)	18 (12.4%)	3 (4.5%)	0.09
Chronic kidney disease	111 (52.4%)	87 (60.0%)	24 (35.8%)	0.001
Previous MI	35 (16.5%)	24 (16.6%)	11 (16.4%)	1.00
Atrial fibrillation	42 (19.8%)	30 (20.7%)	12 (17.9%)	0.71
Severe aortic stenosis	22 (11.0%)	22 (16.1%)	0 (0.0%)	< 0.001
Hemoglobin (mg/dl)	12.8 ± 2.1	12.5 ± 2.2	13.5 ± 1.7	< 0.001
eGFR (ml/min/1.73 m^2^)	54.3 ± 25.4	51.5 ± 27.7	60.4 ± 18.1	0.02
LDL cholesterol (mg/dl)	97.0 ± 31.7	93.9 ± 31.0	104.1 ± 32.3	0.03
*Medications*				
β-blocker	113 (53.3%)	76 (52.4%)	37 (55.2%)	0.77
ACE-i/ARB	121 (57.1%)	85 (58.6%)	36 (53.7%)	0.55
Statin	174 (82.1%)	113 (77.9%)	61 (91.0%)	0.02
Antiplatelet	148 (69.8%)	86 (59.3%)	62 (92.5%)	< 0.001
Anticoagulant	54 (25.5%)	38 (26.2%)	16 (23.9%)	0.87

Values are expressed as mean ± standard deviation or n (%). *ACE-I* angiotensin-converting enzyme inhibitor; *ARB* angiotensin II receptor blocker; *eGFR* estimated glomerular filtration rate; *LDL* low-density lipoprotein; *MI* myocardial infarction

**Table 2:** Physiological findings

	All (*n* = 212)	Deferral group (*n* = 145)	Revascularization group (*n* = 67)	*P* value
Target vessel				0.11
LAD	129 (60.9%)	94 (64.8%)	35 (52.2%)	
LCX	48 (22.6%)	32 (22.1%)	16 (23.9%)	
RCA	35 (16.5%)	19 (13.1%)	16 (23.9%)	
*Hyperemic agents*				0.008
Papaverine	113 (53.3%)	68 (46.9%)	45 (67.2%)	
Nicorandil	99 (46.7%)	77 (53.1%)	22 (32.8%)	
*Physiological findings*				
FFR	0.81 ± 0.05	0.82 ± 0.04	0.77 ± 0.05	< 0.001
FFR ≤ 0.80	105 (49.5%)	50 (34.5%)	55 (82.1%)	< 0.001
RFR	0.89 ± 0.05	0.88 ± 0.05	0.91 ± 0.04	< 0.001
RFR ≤ 0.89	107 (50.5%)	95 (65.5%)	12 (17.9%)	< 0.001

*FFR* fractional flow reserve; *LAD* left anterior descending artery; *LCX* left circumflex artery; *RCA* right coronary artery; *RFR* resting full-cycle ratio

**Table 3:** Clinical outcomes

	All (*n* = 212)	Deferral group (*n* = 145)	Revascularization group (*n* = 67)	*P* value
Follow-up days	406 [217–763]	389 [148–759]	449 [302–764]	0.048
TVF	12 (5.7%)	11 (7.6%)	1 (1.5%)	0.11
Cardiovascular death	4 (1.9%)	3 (2.1%)	1 (1.5%)	1.00
Target vessel MI	1 (0.5%)	1 (0.7%)	0 (0.0%)	1.00
Unplanned revascularization	7 (4.8%)	7 (4.8%)	0 (0.0%)	0.10

*MI* myocardial infarction; *TVF* target vessel failure

**Table 4:** Baseline characteristics after excluding vessels with severe aortic stenosis and hemodialysis

	All (*n* = 173)	Deferral group (*n* = 109)	Revascularization group (*n* = 64)	*P* value
Age (years)	72.2 ± 8.0	73.3 ± 7.8	70.4 ± 8.1	0.02
Men	138 (79.8%)	83 (76.1%)	55 (85.9%)	0.17
Body mass index (kg/m^2^)	23.7 ± 3.8	23.1 ± 3.6	24.7 ± 3.9	0.01
Body surface area (m^2^)	1.69 ± 0.19	1.65 ± 0.18	1.75 ± 0.19	< 0.001
Hypertension	134 (77.5%)	81 (74.3%)	53 (82.8%)	0.26
Diabetes	69 (39.9%)	44 (40.4%)	25 (39.1%)	1.00
Dyslipidemia	125 (72.3%)	80 (73.4%)	45 (70.3%)	0.73
Current smoking	33 (19.1%)	16 (14.7%)	17 (26.6%)	0.07
Chronic kidney disease	78 (45.1%)	57 (52.3%)	21 (32.8%)	0.02
Previous MI	32 (18.5%)	22 (20.2%)	10 (15.6%)	0.55
Atrial fibrillation	36 (20.8%)	24 (22.0%)	12 (18.8%)	0.70
Hemoglobin (mg/dl)	13.1 ± 2.1	12.8 ± 2.3	13.7 ± 1.6	0.006
eGFR (ml/min/1.73 m^2^)	60.0 ± 21.0	58.3 ± 23.9	62.8 ± 14.6	0.18
LDL cholesterol (mg/dl)	96.7 ± 31.1	92.0 ± 29.4	104.7 ± 32.6	0.01
*Medications*				
β-blocker	95 (54.9%)	61 (56.0%)	34 (53.1%)	0.75
ACE-i/ARB	100 (57.8%)	67 (61.5%)	33 (51.6%)	0.21
Statin	152 (87.9%)	93 (85.3%)	59 (92.2%)	0.23
Antiplatelet	130 (75.1%)	70 (64.2%)	60 (93.8%)	< 0.001
Anticoagulant	45 (26.0%)	30 (27.5%)	15 (23.4%)	0.59

Values are expressed as mean ± standard deviation or n (%). *ACE-I* angiotensin-converting enzyme inhibitor; *ARB* angiotensin II receptor blocker; *eGFR* estimated glomerular filtration rate; *LDL* low-density lipoprotein; *MI* myocardial infarction

**Table 5:** Physiological findings after excluding vessels with severe aortic stenosis and hemodialysis

	All (*n* = 173)	Deferral group (*n* = 109)	Revascularization group (*n* = 64)	*P* value
*Target vessel*				0.10
LAD	107 (61.9%)	73 (67.0%)	34 (53.1%)	
LCX	36 (20.8%)	22 (20.2%)	14 (21.9%)	
RCA	30 (17.3%)	14 (12.8%)	16 (25.0%)	
*Hyperemic agents*				0.08
Papaverine	103 (59.5%)	59 (54.1%)	44 (68.8%)	
Nicorandil	70 (40.5%)	50 (45.9%)	20 (31.2%)	
*Physiological findings*				
FFR	0.80 ± 0.05	0.82 ± 0.04	0.76 ± 0.05	< 0.001
FFR ≤ 0.80	102 (59.0%)	47 (43.1%)	55 (85.9%)	< 0.001
RFR	0.90 ± 0.04	0.89 ± 0.04	0.91 ± 0.04	< 0.001
RFR ≤ 0.89	71 (41.0%)	62 (56.9%)	9 (14.1%)	< 0.001

*FFR* fractional flow reserve; *LAD* left anterior descending artery; *LCX* left circumflex artery; *RCA* right coronary artery; *RFR* resting full-cycle ratio

**Table 6:** Clinical outcomes after excluding vessels with severe aortic stenosis and hemodialysis

	All (*n* = 173)	Deferral group (*n* = 109)	Revascularization group (*n* = 64)	*P* value
Follow-up days	397 [208–764]	364 [93–770]	420 [302–764]	0.07
TVF	9 (5.2%)	8 (7.3%)	1 (1.6%)	0.16
Cardiovascular death	4 (2.3%)	3 (2.8%)	1 (1.6%)	1.00
Target vessel MI	1 (0.6%)	1 (0.9%)	0 (0.0%)	1.00
Unplanned revascularization	4 (2.3%)	4 (3.7%)	0 (0.0%)	0.30

*MI* myocardial infarction; *TVF* target vessel failure

## Discussion

This study showed that 212 out of 992 (21.4%) vessels with intermediate coronary lesions had discordant results of FFR and RFR, with the prevalence being approximately 50% for either positive FFR or RFR lesions. Of the 212 vessels, coronary revascularization was deferred in 145 (68.4%) vessels. During the follow-up, TVF was less likely to occur in the revascularized vessels than in the deferral vessels. The cumulative risk of TVF in the deferral group was not significantly different between the vessels with positive FFR and RFR. Our findings indicate that coronary revascularization might reduce the risk of TVF in patients having vessels with discordant results of FFR and NHPRs.

### Discordant physiological indices and outcomes

In the recent international guidelines, both FFR and NHPRs have been recommended as an invasive standard to guide treatment strategies in patients with chronic CAD [1–3]. While FFR ≤ 0.80 and NHPRs ≤ 0.89 are widely recognized as optimal cut-off values for coronary revascularization [1–3], the disagreement between FFR and NHPRs is often found in approximately 20% of lesions with angiographically intermediate epicardial coronary stenosis [8–10], which is in line with our results (i.e., 21.4%). Despite the clinically relevant burden, the clinical outcomes of patients and vessels with the discordant results of FFR and NHPRs have not been fully investigated. In the 3 V FFR-FRIENDS (3-Vessel Fractional Flow Reserve for the Assessment of Total Stenosis Burden and Its Clinical Impact in Patients With Coronary Artery Disease) registry (1024 vessels in 435 patients), the cumulative incidence of vessel-oriented composited outcomes at 5 years in deferred vessels with discordant results of FFR and NHPRs (14.4%) was similar to those in revascularized vessels (14.8%) and was higher than vessels with negative FFR and NHPRs (7.5%) [7], suggesting that the risk of coronary-related events was not small in vessels with the disagreement. Recently, the J-PRIDE registry (Clinical Outcomes of Japanese Patients With Coronary Artery Disease Assessed by Resting Indices and Fractional Flow Reserve: A Prospective Multicenter Registry) (4304 lesions in 3200 patients) confirmed that the deferred revascularization in lesions with discordant FFR and NHPR results led to an increased TVF risk as compared with lesions with concordant negative results [10]. Among the deferred vessels, the 1-year TVF rates were 7.9%, 5.5%, and 1.7% in vessels with positive FFR and negative NHPR, negative FFR and positive NHPR, and negative FFR and NHPR, respectively, whereas no significant differences in clinical outcomes were found among revascularized vessels [10]. In the present study, the cumulative TVF risk in vessels with discordant FFR and RFR results was significantly lower in the revascularization group than in the deferral group, which may confirm the results in the 3 V FFR-FRIENDS and J-PRIDE with the similar incidence of TVF [7, 10]. Although several NHPRs, including iFR, RFR, and diastolic pressure ratio, were included in the large-scale registries, our study exclusively evaluated RFR as an NHPR. Although NHPRs may be used interchangeably [6], RFR is a unique non-hyperemic index in evaluating coronary pressure during the entire cardiac cycle [23]. In a previous study, indeed, the likelihood of being positive (≤ 0.89) was significantly higher in RFR than in diastolic pressure ratio, indicating that all NHPRs may not be equivalent [24]. Therefore, even being confirmatory, we believe that our findings may be clinically relevant. Interestingly, the implementation of coronary revascularization was primarily determined by FFR rather than RFR in the present study. Given that a potential benefit of revascularization over medical therapy alone in reducing TVF was found in vessels with positive FFR and negative NHPR rather than those with negative FFR and positive NHPR in the J-PRIDE registry [10], our clinical decision-making in the present study may have been reasonable. In addition, recent study-level meta-analyses showed that all-cause mortality was increased with revascularization guided by iFR as compared to FFR [25, 26]. Thus, in vessels with discordant results of FFR and NHPR, coronary revascularization may be particularly indicated when FFR is positive, although the present study did not address the differential impact of FFR and RFR on clinical outcomes due to the limited sample size. Taken together, our results and previous reports suggest that coronary revascularization may have the potential to improve clinical outcomes of patients with intermediate epicardial CAD with discordant results of FFR and NHPRs. However, a higher risk of TVF in the deferral rather than revascularization group was mainly driven by an increased risk of unplanned coronary revascularization, and the open-label design may have affected the results. Hence, the possible benefit of coronary revascularization should be interpreted with caution.

### Study limitations

There are some limitations in this study. This was a retrospective, single-center study with a limited sample size. Patients in the revascularization group had older age, lower body mass index, anemia, impaired kidney function, higher probability of severe aortic stenosis, and lower FFR and RFR values, indicating the choice of revascularization strategies in a real-world clinical setting. Thus, the external generalizability of the present study should be carefully considered. The number of events was small to perform multivariable analysis for adjusting potential confounders, even though the stratified analysis with hemodialysis and severe aortic stenosis showed similar results. The implementation of physiological assessment and the choice of therapeutic strategies were left to the discretion of operators. We exclusively focused on RFR in the present study, while other invasive NHPRs and non-wire-based coronary physiological indices were not evaluated [16, 27–30]. In addition, although morphologic patterns of coronary artery stenosis (i.e., focal and diffuse lesions) are reportedly associated with the discordant physiological results and clinical outcomes [31–33], the objective measures of such patterns (e.g., pressure pullback gradient index) were not available in the present study. In the present study, other invasive coronary physiological indices than FFR and RFR were not evaluated [34, 35].

## Conclusion

The discordant results of FFR and RFR were found in approximately 20% of vessels with intermediate epicardial CAD. In such vessels, deferred coronary revascularization was associated with an increased risk of TVF than the revascularization strategy. The possible benefit of coronary revascularization in patients having vessels with discordant results of FFR and NHPRs deserves further large-scale clinical studies.
